# Correction: Prevalence and risk factors for asthma, rhinitis, eczema, and atopy among preschool children in an Andean city

**DOI:** 10.1371/journal.pone.0236843

**Published:** 2020-07-22

**Authors:** Cristina Ochoa-Avilés, Diana Morillo, Alejandro Rodriguez, Philip John Cooper, Susana Andrade, María Molina, Mayra Parra, Andrea Parra-Ullauri, Danilo Mejía, Alejandra Neira, Claudia Rodas-Espinoza, Angélica Ochoa-Avilés

The first author, Cristina Ochoa-Avilés, is incorrectly noted as the corresponding author. The correct corresponding author is Angélica Ochoa-Avilés. Dr. Angélica Ochoa-Avilés’ email address is: angelica.ochoa@ucuenca.edu.ec.
