# Supplementary material for: Prevalence and risk factors for asthma, rhinitis, eczema, and atopy among preschool children in an Andean city
Source: PLoS One. 2020 Jul 10;15(7):e0234633. doi: 10.1371/journal.pone.0234633 (PMC7351199; doi:10.1371/journal.pone.0234633)
Supplement: Attachment — Submitted filename: Response to reviewers.docx [file pone.0234633.s002.docx]

**Rebuttal Letter Article: [PONE-D-20-02044]-[EMID:81850e4da8ff6666]**

**Title: Prevalence and risk factors for asthma, rhinitis, eczema and atopy among preschool children in an Andean city.**

We appreciate the valuable comments. This document is written as follows: reviewer’s

comments are in italic and bold, responses to the comments are preceded by the letter R; extracts of the manuscript are in blue.

**Reviewer's Report:**

***This is an interesting epidemiological study concerning prevalence of Eczema, Asthma and Rhinitis in a Highland pediatric Population of the Andes which seems to be a new Patient collective. Also, it adds some Information, whether higher social income is also a Risk factor in developing countries in the same way as in the industrialized world. Although the study is well written with a highly sophisticated Level of statistical work-up, I gave some very Basic Major remarks mainly concerning the definitions used in this paper.***

***-The high prevalence of eczema patients is astounding and makes me suspicious that the term "eczema" is too vaguely defined in a pediatric Population especially prone to viral infections with a high Risk of developing infection-connected eczema rather than true atopic eczema***.

**R**: We thank the reviewer for this insightful comment. We did observe a high prevalence of eczema in our pediatric population of children aged 3 to 5 years (28%). We used the ISAAC definition for eczema, that has been used widely in epidemiological studies in children and estimated a prevalence similar to that described in other epidemiological studies in Latin America using the same definition. For example, a similar prevalence (22.5%) was estimated among Ecuadorian children aged 6-7 years in the ISAAC Phase III study [(Odhiambo et al. 2009)](https://paperpile.com/c/ckSWXk/YqF14). The differential diagnosis of eczema in 3-5-year olds in this setting include seborrheic dermatitis, scabies and other arthropod bites, miliaria, fungal infections, etc. Recent analyses comparing ISAAC with other definitions of eczema have shown a tendency of the ISAAC definition to overestimate eczema prevalence in tropical compared to sub-tropical settings [(Sánchez, Sánchez, and Cardona 2018)](https://paperpile.com/c/ckSWXk/lcG7). It should be emphasized that the City of Cuenca is sub-tropical where conditions that might inflate ISAAC-based prevalence estimates, such as miliaria and scabies, are less common. We have added now text explaining this to the Discussion (page 14).

***-Also, the term "atopy" is not clearly defined throughout the paper: how do the authors use this term? An atopic parent? A positive Skin prick test?***

**R:** We thank the reviewers comment, the term atopy was defined as “a positive [skin test] reaction to any of the allergens tested” on page 6 of the manuscript following the definition of the European Academy of allergen and clinical immunology (EAACI)[(Johansson et al. 2004)](https://paperpile.com/c/ckSWXk/OpWYq). The definition of atopy is provided also in the abstract: “Atopy was measured by skin prick test (SPT) reactivity to a panel of relevant aeroallergens”. We have clarified the definition of atopy used in this study in the relevant section of the Results on page 9. We acknowledge that the words ‘allergic’ and ‘atopic’ are often used interchangeably so have clarified that ‘allergic diseases’ refers to ‘symptoms of the commonly called ‘allergic diseases”, asthma, rhinitis, eczema, without determination of the actual presence of atopy’ (page 6).

***-Minor remark: Is the Skin prick test series truly representative for the plants abundant in the Highland Andes? They rather seem to be fit for a central European Population completely ignoring endemic plant species in the Highland Ecuador and more General Altiplano Region. Also Information regarding the manufacturer of the test substances is missing.***

**R:** The plant species used for skin prick testing in this study were based on clinical experience and availability of purified extracts. Commercially available extracts representative of the plant species in highland Ecuador or the Andean region are not available. We have added a statement to the limitation paragraph on page 15 discussing this limitation. Information on commercial source of allergens manufacturer has been added to methods (page 6).

***-This was a field study. How did the authors guarantee that allergenicity was not lost by using Skin prick test Solutions that were not stored properly between 4 and 8 Degree Celsius. Especially house dust mite extracts will soon lose their activity through the proteolytic activity of the Major house dust mite allergen components when stored at room temperature for longer time.***

**R:** Allergens were stored at 4-8ºC and aliquots of antigens were transported to the field on ice packs. This sentence has been added to the text on page:

Allergens were stored at 4-8ºC and aliquots of antigens were transported to the field on ice packs.

***-Methods: The authors describe "Scratch test". Hopefully this is only a wrong description and the authors used "Skin prick Tests"?***

**R:** We thank the reviewer for highlighting this error in the text - we did skin prick tests. The word ‘scratch’ has been replaced by ‘prick’. Text now reads: “Allergens were pricked on the forearm and reaction sizes evaluated after 15 minutes” (page 6).

***-Please elaborate more about the rationale about high attitude level (higher than 2,500m) and the atopy.***

**R:** The following text has been added:

Studies in Europe have reported low levels of mite allergens in high altitude settings (>1500 m) related to low humidity [(Custovic et al. 1998; Spieksma, Zuidema, and Leupen 1971)](https://paperpile.com/c/ckSWXk/NgxLo+URczP). Previous studies in Quito, another high altitude city (2,800 m altitude) in the Ecuadorian Andes showed significant levels of indoor dust mites (*D. pteronyssinus* and *D. farinae*) [(Valdivieso, Iraola, and Pinto 2009)](https://paperpile.com/c/ckSWXk/rpNJ) and mite allergens associated with respiratory allergy [(Valdivieso and Iraloa 2011)](https://paperpile.com/c/ckSWXk/sKBj). Data from this study indicate that mites are important sensitizing allergens in pre-school children in another Andean urban setting in a setting with an annual average humidity of which varies seasonally (average 83.7%, range 77.5-89.4%) [(Quinteros Espinoza and Ramírez Parra 2018)](https://paperpile.com/c/ckSWXk/ec1MJ).

***-Regarding the logistic regression analysis, the value of each variable should be display also. By showing only the percentage and Odds ratio are not enough for determining the degree of association of risk factors.***

**R:** The values of each variable are displayed in the logistic regression analysis in table 4. The table has been modified as follows:

| ***Predictors*** | ***Asthma (N=95/535)*** | | | ***Rhinitis (N=257/535)*** | | | ***Eczema (N=150/535)*** | | | ***Atopy (N=179/535)*** | | |
| --- | --- | --- | --- | --- | --- | --- | --- | --- | --- | --- | --- | --- |
|  | Frecuency | Bivariate | Multivariate | Frecuency | Bivariate | Multivariate | Frecuency | Bivariate | Multivariate | Frecuency | Bivariate | Multivariate |
|  | N (%) | OR (95% CI)  P Value | OR (95% CI)  P Value | N (%) | OR (95% CI)  P Value | OR (95% CI)  P Value | N (%) | OR (95% CI)  P Value | OR (95% CI)  P Value | N (%) | OR (95%CI)  P Value | OR (95% CI)  P Value |
| *Socioeconomic status* | | | | | | | | | | | | |
| Low | 13 (13.7) | 1 |  | 35 (13.6) | 1 |  | 33 (22.0) | 1 |  | 29 (16.2) | 1 |  |
| Medium | 47 (49.5) | 1.83 (1.08-3.12) 0.03 | 1.42 (0.71-2.83)  0.31 | 114 (44.4) | 1.96 (1.22-3.15) 0.07 | **1.75 (0.97-3.13)**  **0.06** | 80 (53.3) | 1.18 (0.69-2.00) 0.52 |  | 88 (49.2) | 1.63 (1.09-2.45) 0.02 | 1.17 (0.92-1.48) 0.20 |
| High | 32 (33.7) | 1.76 (0.91-3.37) 0.08 | 0.90 (0.38-2.10) 0.80 | 99 (38.5) | 3.07 (1.79-5.25) <0.001 | **2.09 (1.10-3.96)**  **0.03** | 33 (22.0) | 0.57 (0.28-1.14) 0.10 |  | 58 (32.4) | 1.47 (0.88-2.47) 0.13 | 1.47 (0.88-2.47) 0.13 |
| *Type of school* | | | | | | | | | | | | |
| *Public* | 43 (45.3) |  |  | 130 (50.6) |  |  | 92 (61.3) |  |  | 88 (49.2) |  |  |
| *Private* | 52 (54.7) | 1.56 (1.00-2.45)  0.05 |  | 127 (49.4) | 1.34 (0.91-1.98) 0.13 |  | 58 (38.6) | 0.67 (0.43-1.07) 0.09 | 0.80 (0.43-1.48) 0.47 | 91 (50.8) | 1.37 (0.96-1.97) 0.08 | 1.43 (0.96-2.13)  0.07 |
| *Bathroom* | | | | | | | | | | | | |
| *Hygienic service* | 94 (99.0) |  |  | 251 (97.7) |  |  | 148 (98.6) |  |  | 171 (95.5) |  |  |
| *Latine or field)* | 1.0 (1.1) | 0.41 (0.07-2.58) 0.33 |  | 4 (1.6) | 0.53 (0.24-1.21) 0.13 |  | 1 (0.6) | 0.23 (0.04-1.34) 0.10 | 0.49 (0.11-2.11)  0.34 | 6 (3.3) | 2.04 (0.54-7.61) 0.29 |  |
| *Household appliances* | | | | | | | | | | | | |
| *2 or less* | 18 (19.0) |  |  | 47 (14.4) |  |  | 28 (18.6) |  |  | 32 (17.9) |  |  |
| *3 or more* | 77 (81.1) | 0.93 (0.67-1.29) 0.67 |  | 220 (85.7) | 1.30 (0.97-1.74) 0.078 | 1.04 (0.75-1.44)  0.79 | 122 (81.2) | 0.78 (0.6-1.00) 0.05 | 0.97 (0.67-1.41)  0.89 | 147 (82.1) | 0.92 (0.74-1.15) 0.46 |  |
| *Birth order* | | | | | | | | | | | | |
| *1-2* | 18 (19.0) |  |  | 45 (17.5) |  |  | 31 (20.6) |  |  | 33 (18.4) |  |  |
| *3 or more* | 77 (81.1) | 0.90 (0.47-1.69) 0.73 |  | 212 (82.5) | 1.01 (0.67-1.53) 0.95 |  | 118 (78.6) | 0.74 (0.52-1.06) 0.10 | 0.74 (0.43-1.29)  0.28 | 145 (81.0) | 0.91 (0.57-1.45)  0.69 |  |
| *Breastfeeding (months)* | | | | | | | | | | | | |
| *< 6* | 14 (14.7) |  |  | 47 (18.3) |  |  | 22 (14.6) |  |  | 30 (16.8) |  |  |
| *6-12* | 30 (31.6) |  |  | 92 (35.8) |  |  | 48 (32.0) |  |  | 63 (35.2) |  |  |
| *12-24* | 33(34.7) |  |  | 71 (27.6) |  |  | 47 (31.3) |  |  | 60 (33.5) |  |  |
| *>24* | 14 (14.7) | 1.19 (0.94-1.51) 0.14 |  | 30 (11.7) | 0.90 (0.76-1.07) 0.22 |  | 24 (16.0) | 1.22 (1.01-1.49) 0.04 | 1.05 (0.79-1.39)  0.72 | 17 (9.5) | 0.96 (0.78-1.20)  0.74 |  |
| *Daycare* | | | | | | | | | | | | |
| *Yes* | 35 (36.8) |  |  | 119 (46.3) |  |  | 49 (32.6) |  |  | 63 (35.2) |  |  |
| *No* | 60 (63.2) | 1.06 (0.68-1.65) 0.78 |  | 136 (52.9) | 0.49 (0.37-0.64) 0.001 | **0.64 (0.46-0.88)**  **0.009** | 101 (67.3) | 1.38 (0.88-2.16) 0.16 |  | 114 (63.7) | 1.16 (0.77-1.77)  0.48 |  |
| *Mother smoking* | | | | | | | | | | | | |
| *Yes* | 2 (2.1) |  |  | 11 (4.3) |  |  | 6 (4.0) |  |  | 3 (1.7) |  |  |
| *No* | 93 (97.9) | 0.50 (0.14-1.78) 0.27 |  | 244 (94.9) | 1.34 (0.61-2.92) 0.47 |  | 142 (94.6) | 1.11 (0.39-3.22) 0.84 |  | 133 (96.6) | 0.34 (0.12-0.96) 0.04 | 0.39 (0.11-1.34)  0.13 |
| *Family smoking* | | | | | | | | | | | | |
| *Yes* | 19 (21.0) |  |  | 47 (18.3) |  |  | 34 (22.6) |  |  | 22 (12.3) |  |  |
| *No* | 75 (79.0) | 1.26 (0.65-2.45) 0.47 |  | 207 (80.5) | 1.17 (0.69-2.00) 0.56 |  | 115 (76.6) | 1.65 (1.12-2.44) 0.01 | 1.30 (0.75-2.24)  0.32 | 156 (87.2) | 0.57 (0.36-0.90) 0.01 | 0.67 (0.38-1.16)  0.15 |
| *Dog (inside house)* | | | | | | | | | | | | |
| *No* | 63 (66.3) |  |  | 185 (71.9) |  |  | 103 (68.6) |  |  | 137 (76.5) |  |  |
| *Yes* | 32 (33.7) | 0.82 (0.49-1.37) 0.43 |  | 72 (28.0) | 1.22 (0.82-1.80) 0.33 |  | 47 (31.3) | 0.93 (0.62-1.38) 0.70 |  | 42 (23.5) | **1.64 (1.11-2.44) 0.01** | **1.67 (1.05-2.66)**  **0.03** |
| *Maternal rhinitis* | | | | | | | | | | | | |
| *No* | 38 (56.8) |  |  | 154 (60.0) |  |  | 43 (28.6) |  |  | 125 (69.8) |  |  |
| *Yes* | 54 (40.0) | 2.21 (1.38-3.55) 0.002 | **1.85 (1.01-3.41)**  **0.04** | 92 (35.8) | **2.75 (1.79-4.22) 0.001** | **2,29 (1.31-3.98)**  **0,005** | 98 (28.6) | 1.25 (0.78-2.01) 0.35 |  | 46 (25.8) | 0.98 (0.67-1.42) 0.91 |  |
| *Maternal eczema* | | | | | | | | | | | | |
| *No* | 18 (18.9) |  |  | 201 (78.2) |  |  | 108 (72.0) |  |  | 145 (81.0) |  |  |
| *Yes* | 69 (72.3) | 2.03 (0.96-4.28) 0.064 | 1.58 (0.68-3.79)  0.27 | 38 (14.8) | 1.63 (0.84-3.17) 0.15 |  | 28 (18.6) | 2.28 (1.42-3.68) 0.001 | 1.73 (0.91-3.28)  0.08 | 25 (13.9) | 1.25 (0.72-2.15) 0.43 |  |
| *Paternal rhinitis* | | | | | | | | | | | | |
| *No* | 21 (22.1) |  |  | 158 (61.5) |  |  | 90 (60.0) |  |  | 114 (63.7) |  |  |
| *Yes* | 58 (61.1) | 1.22 (0.68-2.17) 0.49 |  | 68 (26.5) | 2.03 (1.34-3.07) 0.001 | 1.16 (0.67-2.00)  0.58 | 35 (23.3) | 1.39 (0.88-2.19) 0.16 |  | 39 (21.8) | 1.17 (0.75-1.84)  0.48 |  |
| *Paternal eczema* | | | | | | | | | | | | |
| *No* | 68 (71.6) |  |  | 200 (77.8) |  |  | 101 (67.3) |  |  | 143 (80.0) |  |  |
| *Yes* | 9 (9.5) | 1.69 (0.76-3.75) 0.186 |  | 25 (9.7) | **2.35 (1.30-4.26) 0.005** | **2.07** **(1.11-3.86)**  **0.02** | 21 (14.0) | **4.22 (2.30-7.75) <0.001** | **3.73** **(1.51-9.20)**  **0.07** | 11 (6.1) | 0.84 (0.38-1.84) 0.65 |  |

**References**

[*Custovic, A., A. Simpson, M. D. Chapman, and A. Woodcock. 1998. “Allergen Avoidance in the Treatment of Asthma and Atopic Disorders.” Thorax 53 (1): 63–72.*](http://paperpile.com/b/ckSWXk/NgxLo)

[*Johansson, S. G. O., Thomas Bieber, Ronald Dahl, Peter S. Friedmann, Bobby Q. Lanier, Richard F. Lockey, Cassim Motala, et al. 2004. “Revised Nomenclature for Allergy for Global Use: Report of the Nomenclature Review Committee of the World Allergy Organization, October 2003.” The Journal of Allergy and Clinical Immunology 113 (5): 832–36.*](http://paperpile.com/b/ckSWXk/OpWYq)

[*Odhiambo, Joseph A., Hywel C. Williams, Tadd O. Clayton, Colin F. Robertson, M. Innes Asher, and ISAAC Phase Three Study Group. 2009. “Global Variations in Prevalence of Eczema Symptoms in Children from ISAAC Phase Three.” The Journal of Allergy and Clinical Immunology 124 (6): 1251–58.e23.*](http://paperpile.com/b/ckSWXk/YqF14)

[*Quinteros Espinoza, María Eugenia, and Omar Emmanuel Ramírez Parra. 2018. “Modelamiento de La Precipitación En La Zona Urbana de La Ciudad de Cuenca.” dspace.ups.edu.ec.*](http://paperpile.com/b/ckSWXk/ec1MJ) [*http://dspace.ups.edu.ec/handle/123456789/15799*](http://dspace.ups.edu.ec/handle/123456789/15799)[*.*](http://paperpile.com/b/ckSWXk/ec1MJ)

[*Sánchez, Jorge, Andrés Sánchez, and Ricardo Cardona. 2018. “Critical Review of ISAAC Results for Atopic Dermatitis in Tropical Cities.” Revista Alergia Mexico 65 (4): 389–99.*](http://paperpile.com/b/ckSWXk/lcG7)

[*Spieksma, F. T., P. Zuidema, and M. J. Leupen. 1971. “High Altitude and House-Dust Mites.” British Medical Journal 1 (5740): 82–84.*](http://paperpile.com/b/ckSWXk/URczP)

[*Valdivieso, R., and V. Iraloa. 2011. “Monthly Variation of Dermatophagoides Allergens and Its Influence on Respiratory Allergy in a High Altitude Environment (Quito, 2800 M Asl in Andean Ecuador).” Allergologia et Immunopathologia 39 (1): 10–16.*](http://paperpile.com/b/ckSWXk/sKBj)

[*Valdivieso, R., V. Iraola, and H. Pinto. 2009. “Presence of Domestic Mites at an Extremely High Altitude (4800 M) in Andean Ecuador.” Journal of Investigational Allergology & Clinical Immunology: Official Organ of the International Association of Asthmology and Sociedad Latinoamericana de Alergia E Inmunologia 19 (4): 323–24.*](http://paperpile.com/b/ckSWXk/rpNJ)
