# Supplementary material for: Prevalence and risk factors for asthma, rhinitis, eczema, and atopy among preschool children in an Andean city
Source: PLoS One. 2020 Jul 10;15(7):e0234633. doi: 10.1371/journal.pone.0234633 (PMC7351199; doi:10.1371/journal.pone.0234633)
Supplement: Attachment — Submitted filename: Response to reviewers.docx [file pone.0234633.s003.docx]

**Rebuttal Letter Article: [PONE-D-20-02044]-[EMID:81850e4da8ff6666]**

**Title: Prevalence and risk factors for asthma, rhinitis, eczema, and atopy among preschool children in an Andean city.**

We appreciate the valuable comments. This document is written as follows: reviewer’s

comments are in italic and bold, responses to the comments are preceded by the letter R; extracts of the manuscript are in blue.

**Reviewer's Report:**

***There is an error in the fourth sentence in the discussion section. After "and eczema (28.0%)" you should have a comma. Please check carefully for other errors.***

R: we appreciate the careful revision that has been made to the manuscript. We corrected the error in the fourth sentence of the discussion.

“… symptoms of asthma (17.8%), rhinitis (48.0%), and eczema (28.0%), only a small fraction of…”

***You need to check for grammatical and typographical errors in the text.***

R: grammatical and typographical errors were carefully checked trough the manuscript. All the modifications made were highlighted in the text in blue.
